# Multimodality Imaging for Facilitating Percutaneous Coronary Intervention of a Calcified Nodule Post Redo TAVR

**DOI:** 10.1016/j.jaccas.2025.104013

**Published:** 2025-06-25

**Authors:** Arif Al Nooryani, Rania Taha, George Sianos, Ahmed Khashaba, Basem Baradie, Nagwa Abdelrahman

**Affiliations:** a Cardiovascular Department, Al Qassimi Hospital, Emirates Health Services, Sharjah, United Arab Emirates; b Emirates Health Services, Al Qassimi Hospital, Sharjah, United Arab Emirates; c Department of Cardiology and Cardiovascular Diseases, Faculty of Medicine, Ain Shams University, Cairo, Egypt; d Department of Cardiovascular Medicine, Faculty of Medicine, Assiut University, Assiut, Egypt

**Keywords:** aortic valve, computed tomography, intravascular ultrasound, percutaneous coronary intervention

## Abstract

**Background:**

Transcatheter aortic valve replacement (TAVR) is vital for treating aortic stenosis, but coronary access after redo TAVR remains challenging. As TAVR is extended to younger patients, managing concurrent coronary artery disease will become crucial, with multidetector computed tomography (MDCT) playing a key role in coronary intervention planning.

**Case Summary:**

An 83-year-old woman with multiple comorbidities and a history of redo TAVR and recent left main coronary artery stenting presented with NSTEMI. Imaging showed significant coronary involvement, prompting MDCT and intravascular ultrasound (IVUS) evaluation. Percutaneous coronary intervention (PCI) was performed, successfully treating severe calcified ostial stenosis.

**Discussion:**

This case demonstrates the value of MDCT and IVUS in managing complex coronary lesions after redo TAVR. It highlights the importance of preprocedural imaging to overcome coronary access challenges in patients with previous TAVR.

**Take-Home Messages:**

MDCT and IVUS are vital for planning PCI in post redo-TAVR patients. Coronary access is achievable with proper imaging guidance.

## Visual Summary

**Figure:**
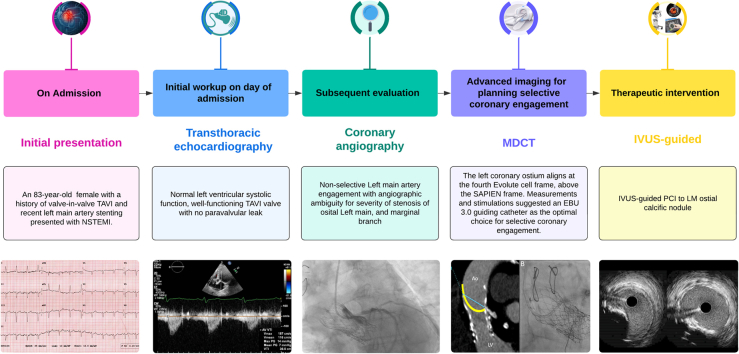

## Past Medical History

An 83-year-old woman with type 2 diabetes, hypertension, dyslipidemia, ischemic heart disease, and chronic kidney disease presented with non–ST-segment elevation myocardial infarction (NSTEMI). The patient has a history of severe aortic stenosis (AS) treated with transcatheter aortic valve replacement (TAVR) with an Edwards SAPIEN 3 valve (Edwards Lifesciences) through surgical direct aortic access (ministernotomy) in 2017 and redo TAVR with the Medtronic Evolut PRO system (Medtronic) through femoral access in July 2024 to treat recurrence of the AS. One month later, percutaneous coronary intervention (PCI) of the left main (LM) coronary artery with stent implantation of a drug-eluting stent (DES) in the direction of the left circumflex (LCx) artery and crossover of the left anterior descending (LAD) artery ostium was performed.

**Take-Home Messages**

• Multimodality imaging, including MDCT and IVUS, is essential for accurate preprocedural planning and overcoming coronary access challenges in patients with previous TAVRs, particularly in the context of complex coronary lesions.
• Mastery of these imaging techniques can significantly improve the success of coronary interventions, by guiding effective decision making and ensuring optimal outcomes in patients requiring PCI after TAVR.

## History of Presentation

On admission, the patient was vitally stable and alert, and she had minimal chest pain. Physical examination revealed a systolic murmur at the aortic area. A 12-lead electrocardiogram showed normal sinus rhythm, an incomplete left bundle branch block, left-axis deviation, and poor R-wave progression. The patient’s troponin I levels increased from 967 ng/L on admission to 2,885 ng/L after a total of 5 hours. The patient had a glomerular filtration rate (GFR) of 37 mL/min/1.73 m^2^ and a normal creatinine level.

## Differential Diagnosis

The primary differential diagnosis for this patient was NSTEMI. Given the recent LM artery stenting, in-stent restenosis or thrombosis was a potential cause of the elevated troponins and chest pain. Additionally, the presence of multiple cardiovascular risk factors further increased the likelihood of progressive atherosclerotic disease contributing to myocardial ischemia. Another consideration was the presence of valvular heart disease–related symptoms. The patient had a history of severe AS that was treated with TAVR twice. Although redo TAVR generally results in hemodynamic improvement, the possibility of valve dysfunction, prosthetic valve thrombosis, or paravalvular regurgitation leading to myocardial strain and subsequent troponin elevation warranted evaluation. The presence of a systolic murmur on examination raised concerns about residual valvular disease.

## Investigations

**Figure 1:**
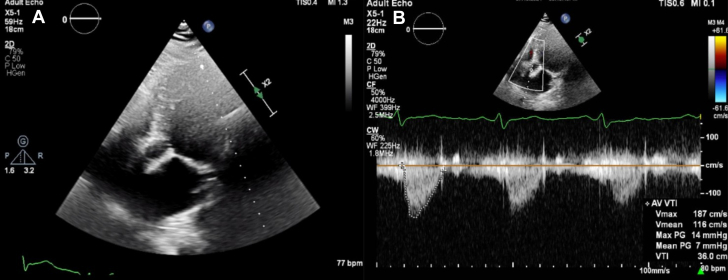
Transthoracic Echocardiography of Redo Transcatheter Aortic Valve Replacement (A) A 2-dimensional (2D) apical 5-chamber view demonstrates the left ventricular outflow tract and the aortic valve (AV), with the previous transcatheter heart valves appearing as echogenic structures. (B) Doppler imaging across the aortic valve confirms proper function of the previous transcatheter heart valves, with no evidence of stenosis or regurgitation. The recorded peak velocity is 187 cm/s, the mean pressure gradient (PG) is 7 mm Hg, and the velocity-time integral (VTI) is 36.0 cm.

**Figure 2:**
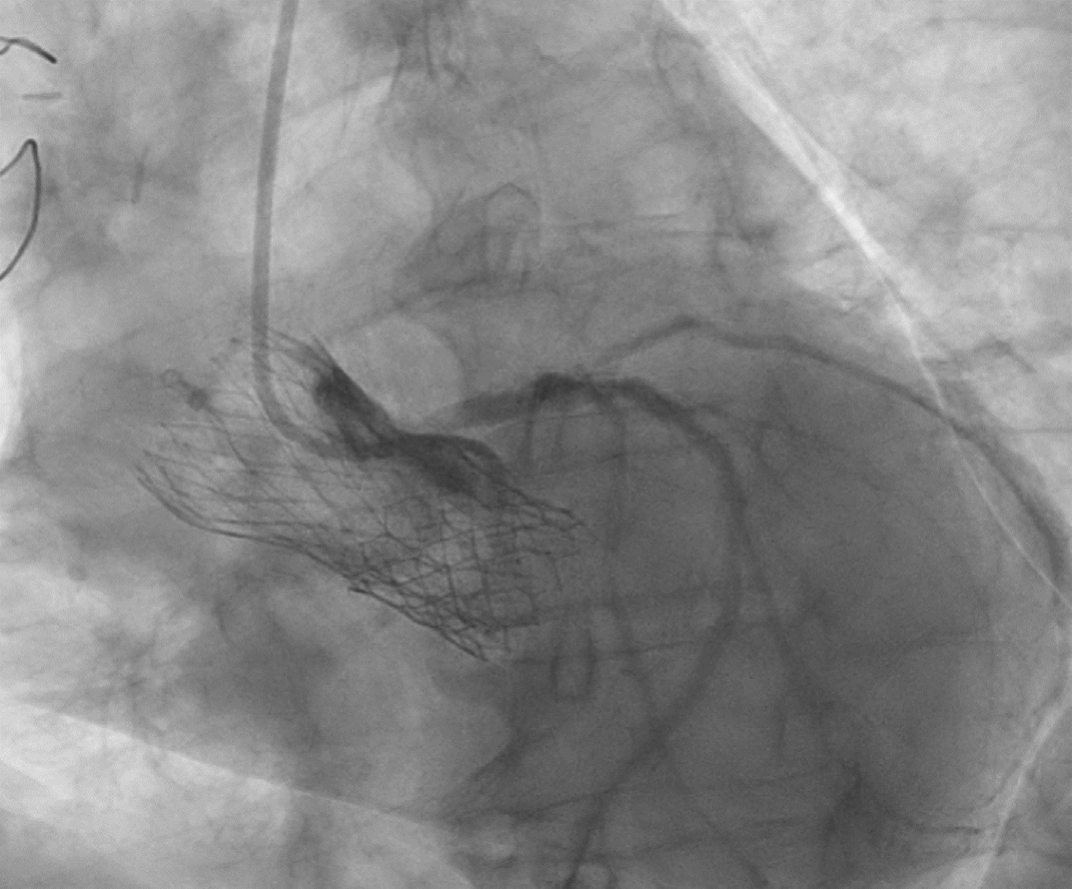
Nonselective Left Coronary Angiography Cardiac catheterization showing the frame of both transcatheter aortic valve implants: the outer SAPIEN 3 valve (Edwards Lifesciences), and the inner Evolut PRO valve (Medtronic). Nonselective left main artery engagement resulting in angiographic ambiguity for severity of stenosis of the ostial left main artery and marginal branch.

Transthoracic echocardiography showed normal left ventricular systolic function, grade 1 diastolic dysfunction, no regional wall motion abnormality, and a well-functioning transcatheter heart valve (THV) with no paravalvular leak (Figures 1A and 1B, Videos 1 and 2). Diagnostic coronary angiography (CAG) through the right femoral artery was performed. The LM artery could not be selectively engaged using either the Judkins left 4.0 catheter or the internal mammary artery catheter. Given the patient’s borderline GFR, further catheterization attempts were avoided. Instead, a decision was made to proceed with multidetector computed tomography (MDCT) for precise localization of the LM artery and optimal procedural planning. The coronary arteries were heavily calcified. There was angiographic ambiguity for severe LM ostial stenosis and severe calcific stenosis of the marginal branch (Figure 2, Video 3). The LAD, LCx, and right coronary arteries appeared free from any significant lesions.

MDCT was performed for the following purposes:

1. Describing the morphologic features of the THVs and their spatial relationships: The outer valve was a balloon-expandable SAPIEN 3 (23-mm) valve, and the inner valve was a self-expanding Evolut PRO (26-mm) valve. The SAPIEN valve leaflets were contracted and thickened; its metallic frame aligns with the midsection of the third Evolut node (Figure 3A).

**Figure 3:**
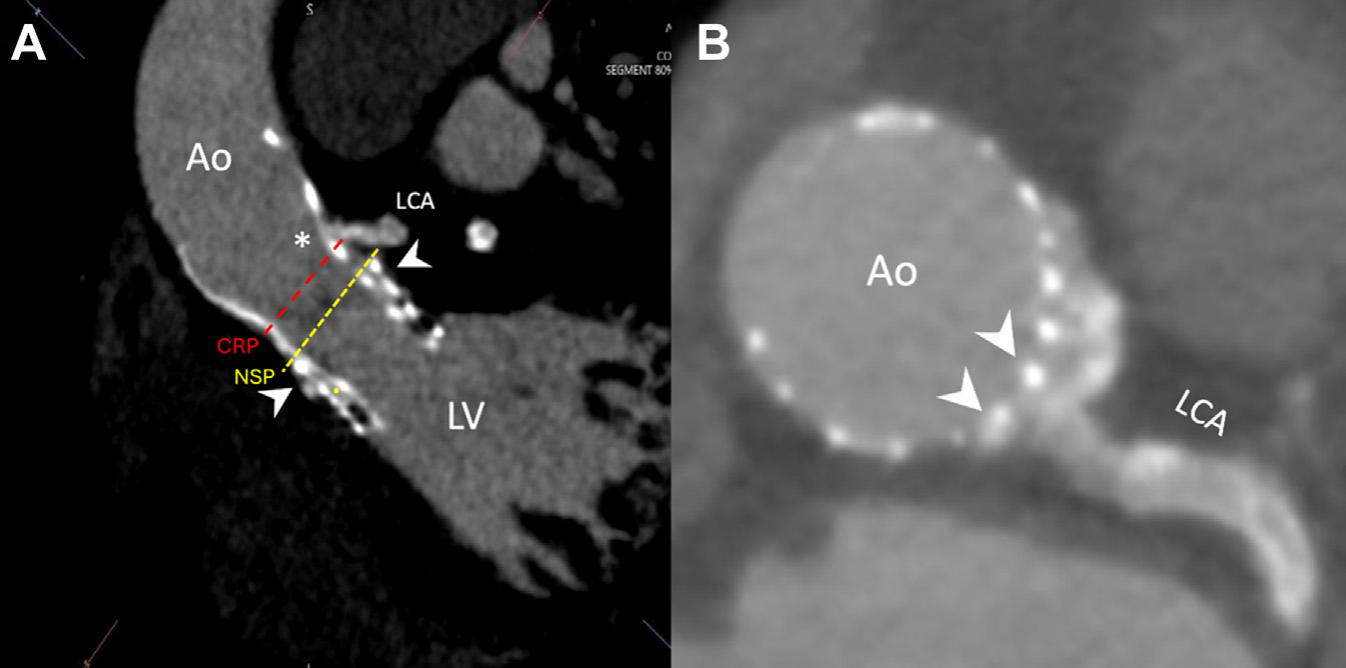
Multidetector Computed Tomography Morphologic Features (A) Parasagittal multidetector computed tomography imaging illustrates the spatial relationship between the previously implanted transcatheter heart valves and the coronary origins. The SAPIEN valve (Edwards Lifesciences) is depicted as the larger outer stent, and the inner Evolut PRO valve (Medtronic) is nested within the SAPIEN, with minimal separation between the 2 valves. The left coronary artery (asterisk) originates above the upper boundary of the SAPIEN metallic frame (arrowheads) at the midlevel of the fourth Evolut cell frame. The neoskirt plane (NSP) of the SAPIEN valve (dashed yellow line) is identified below the coronary risk plane (CRP) of the left coronary artery (dashed red line). The yellow dot marks the contracted leaflets of the SAPIEN valve. (B) Multidetector computed tomography axial angulation demonstrating that the LCA originates within a free cell space (arrowheads). Ao = aorta; LCA = left coronary artery; LV = left ventricle.
2. Evaluating coronary access risk: First, the neoskirt plane (NSP) of the Sapien 3 valve was identified, which was below the coronary risk plane for the left coronary artery (LCA), thus not participating in the difficulty of LM cannulation (Figure 3A). The Evolut PRO valve-to-aorta (VTA) distance was 2.5 mm, whereas the valve-to-coronary (VTC) distance was 2.8 mm, the sinotubular junction (STJ) width was 24.4 mm, the valve-to-SJT distance was 1.5 mm, and the Evolut commissure-coronary angle measured 24.8° (Figures 4A to 4D). Notably, despite minor malalignment of the Evolut valve (commissural-coronary angle, 24.8°), computed tomography (CT) indicated that the LM artery origin opposed a free node space. The proximal 2 to 3 mm of the LM artery was not covered by the previously implanted stent, thus negating any possible interaction with the Evolut metallic frame. A calcified nodule was identified in the proximal segment, likely representing an extension of aortic root calcification (Figure 3B).

**Figure 4:**
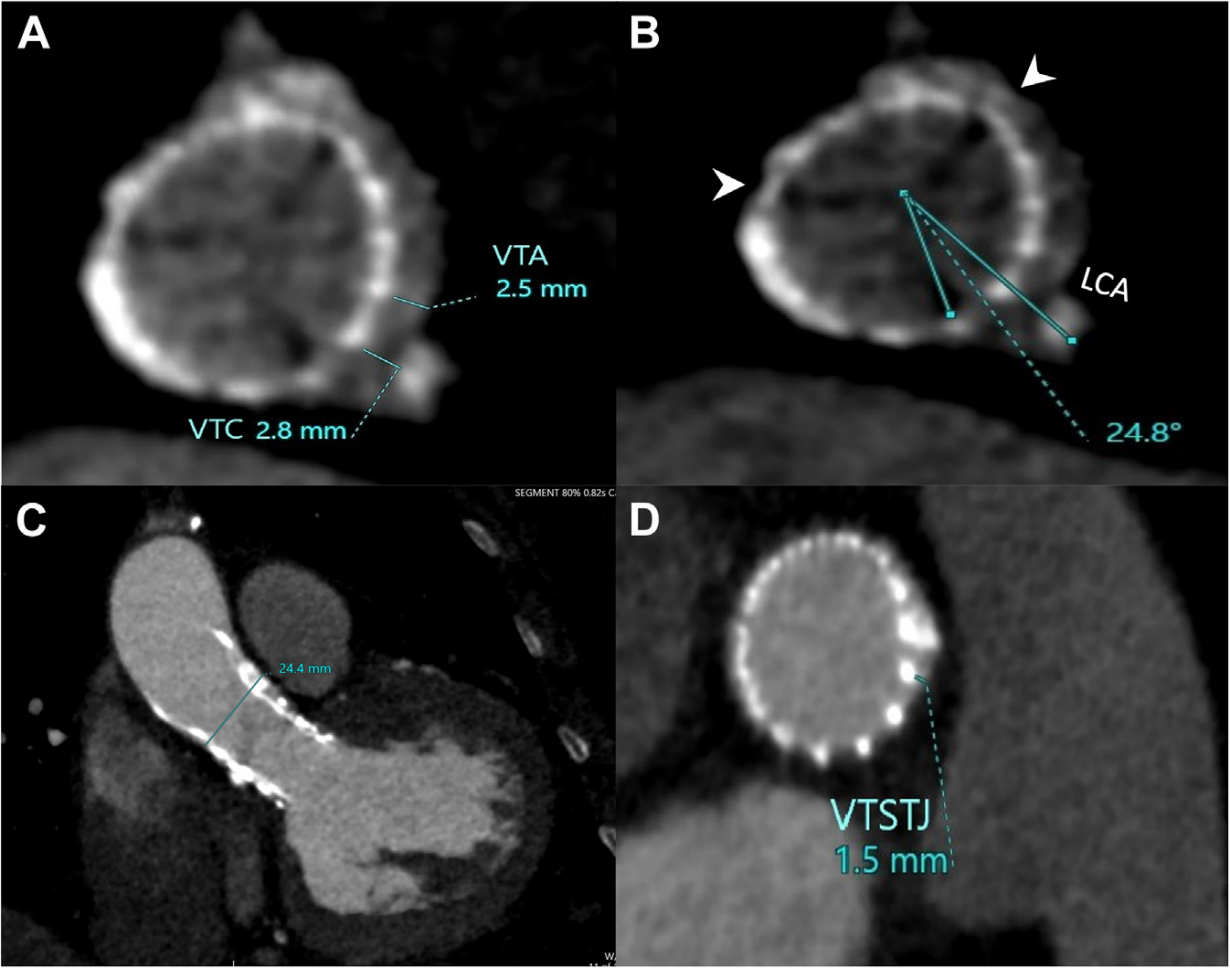
Multidetector Computed Tomography–Driven Measurements (A) The Evolut PRO (Medtronic)–to-aorta distance is 2.5 mm, the Evolut PRO–to-coronary distance is 2.8 mm, (B) the commissural-coronary angle measures 24.8° (arrowheads highlight the neocommissures), (C) the sinotubular junction width is 24.4mm, and (D) the valve-to-sinotubular junction (VTSTJ) distance is 1.5 mm. VTA = valve to aorta; VTC = valve to coronary.
3. Planning the PCI procedure: The LCA origin was detected at the midlevel of the fourth Evolut node, 1 node above the upper border of the SAPIEN metallic frame. MDCT analysis was used to determine the optimal projection for LM artery cannulation, which was confirmed to be left anterior oblique (LAO) 40° with a cranial angulation of 15° (Figures 5A and 5B).

**Figure 5:**
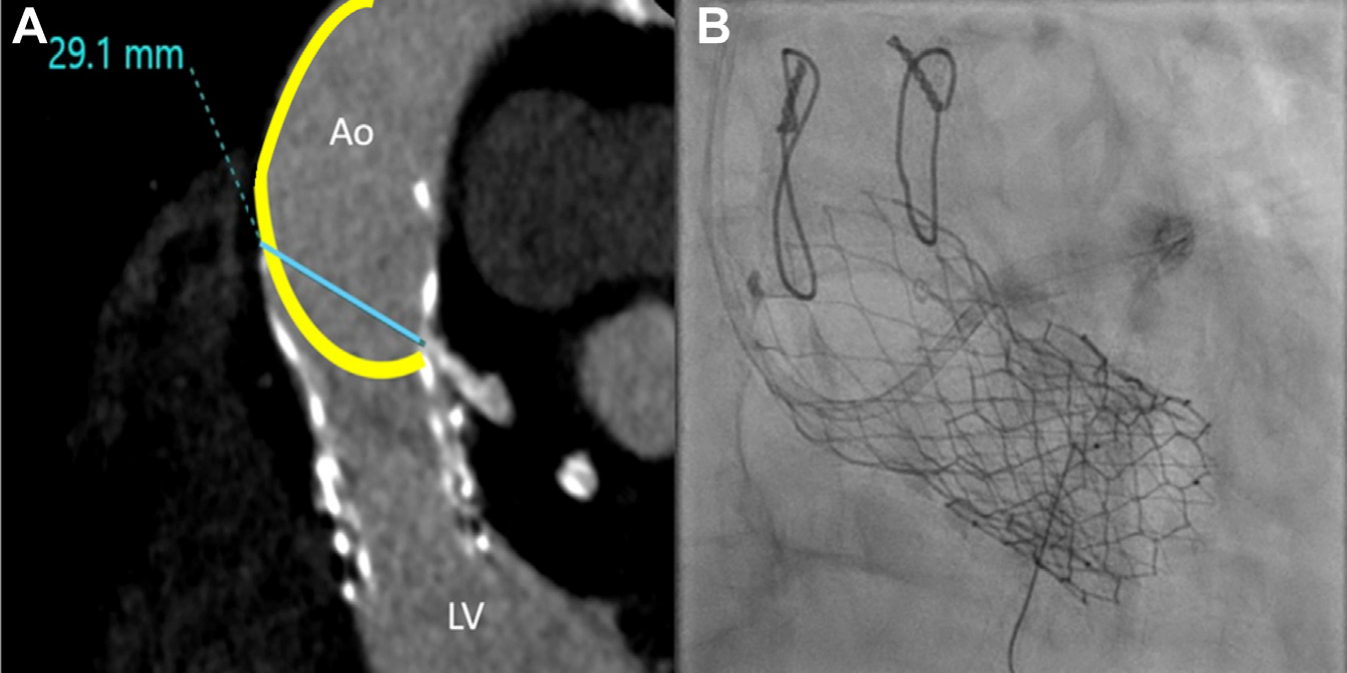
Multidetector Computed Tomography Planning for Guiding Catheter Selection (A) Multidetector computed tomography simulation of the guiding catheter shows the recreated catheter’s tip (yellow curve) positioned 2.9 cm from the greater curvature of the aortic wall adjacent to the left main artery origin, proposing an extra backup (EBU) 3.0 guiding catheter as the optimal choice for selective coronary engagement. (B) Fluoroscopic imaging demonstrates the EBU 3.0 catheter, selected on the basis of multidetector computed tomography measurements, successfully engaging the left coronary artery. Abbreviations as in Figure 3.

Guiding catheter selection was planned on the basis of CT imaging; the distance between the greater curvature of the aortic wall facing the LM artery origin and the stent frame opposite the LM ostium was measured in a curved trajectory simulating guiding catheters. The extra backup (EBU) 3.0 guiding catheter was found to be the optimal choice for coronary engagement according to the CT morphologic and geometric findings (Figures 5A and 5B).

## Management

**Figure 6:**
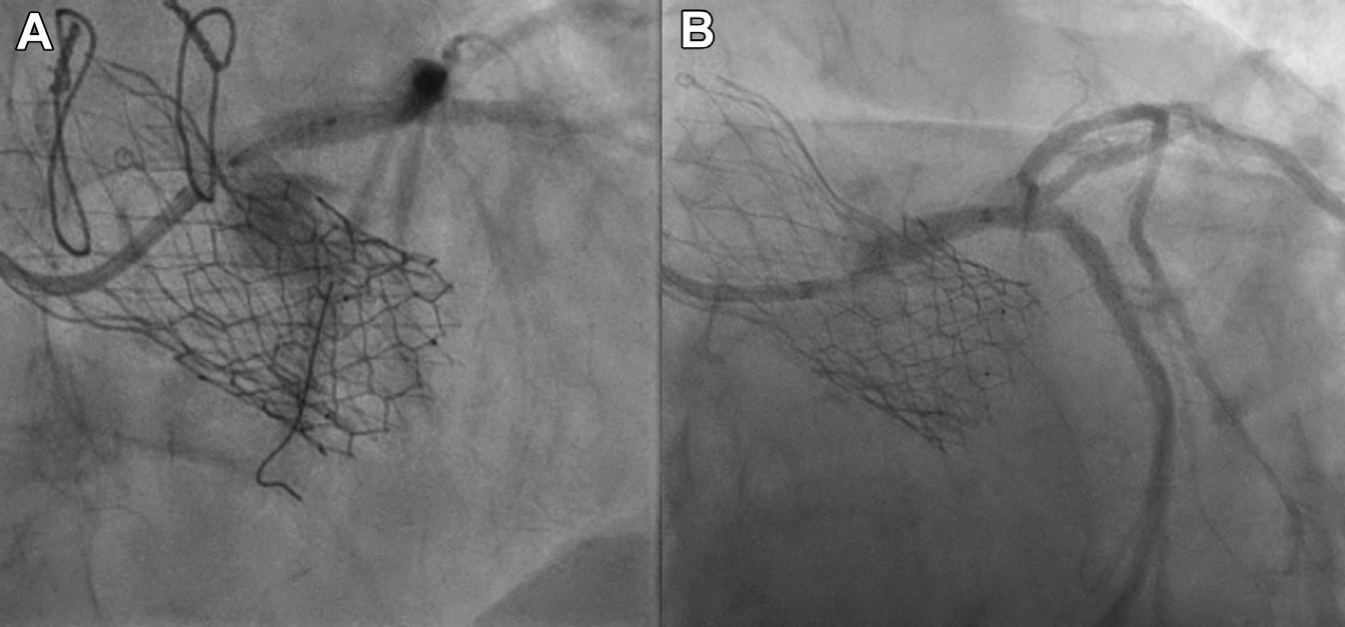
Left Coronary Artery Revascularization (A) Fluoroscopic imaging depicts stent positioning at the left coronary artery ostium in relation to the transcatheter valve prostheses. (B) Poststent deployment angiography confirms restored coronary flow and resolved stenosis, with the deployed stent maintaining left coronary artery patency. The images emphasize the challenges of percutaneous coronary intervention in this setting and the importance of precise stent positioning with multimodal guidance.

**Figure 7:**
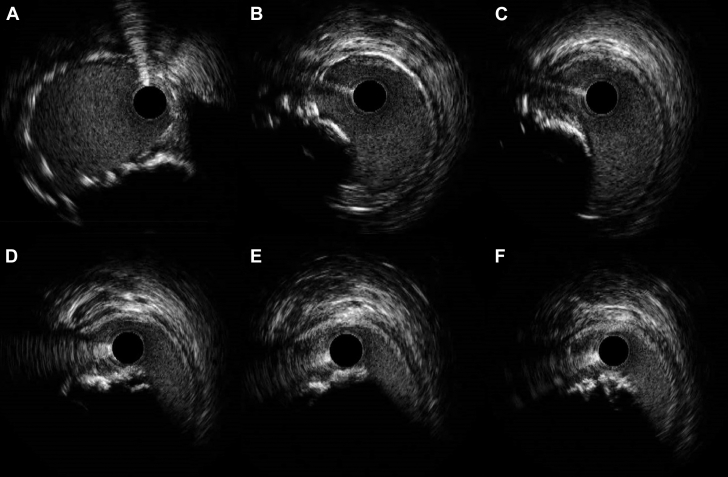
Sequential Intravascular Ultrasound Imaging of Left Main Artery (A) Intravascular ultrasound image demonstrates a patent stent in the left main artery. (B to F) Sequential intravascular ultrasound images reveal an uncovered proximal left main segment with an eccentric calcified nodule protruding into the vessel lumen and causing significant stenosis.

**Figure 8:**
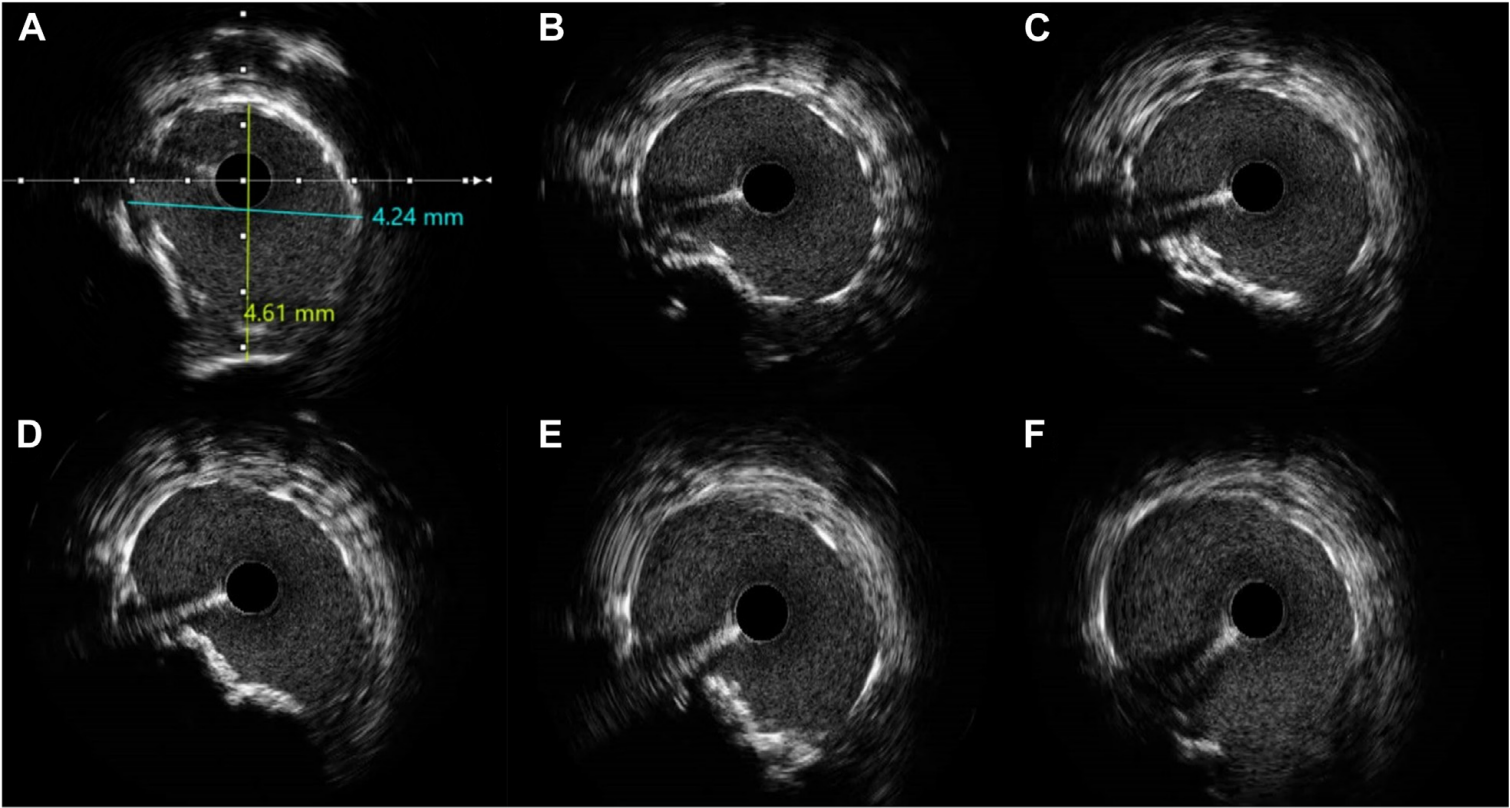
Intravascular Ultrasound-Guided Left Main Artery Stenting (A) Intravascular ultrasound image of left main artery stent with a measurement of 4.2 mm × 4.6 mm, guided post-dilatation by a 5-mm noncompliant balloon. (B to F) Sequential intravascular ultrasound images post-stent optimization reveal good stent expansion and proper apposition of left main artery stent.

PCI was performed through the right radial approach. An EBU 3.0 guiding catheter was manipulated in the LAO 40° cranial 15° projection to target 1 Evolut node above the upper border of the SAPIEN frame (Figure 5B, Video 4), and successful crossing of the LM artery was achieved using a Runthrough guidewire (Terumo) (Figure 6A). Intravascular ultrasound (IVUS) imaging revealed a clearly patent stent in the LM and proximal LCx arteries (Figure A); however, the ostium of the LM artery was not covered and showed 90% ostial calcified nodular stenosis (Figures 7B to 7F, Video 5). PCI with stent implantation was performed using a Xience (Abbott) 4.0 mm × 12 mm DES (Figure 6A). Post-dilatation was guided by IVUS using 4.5-mm and 5.0-mm balloons, resulting in excellent final angiographic (Figure 6B) and IVUS outcomes (Figures 8A to 8F, Video 6).

**Visual Summary:**
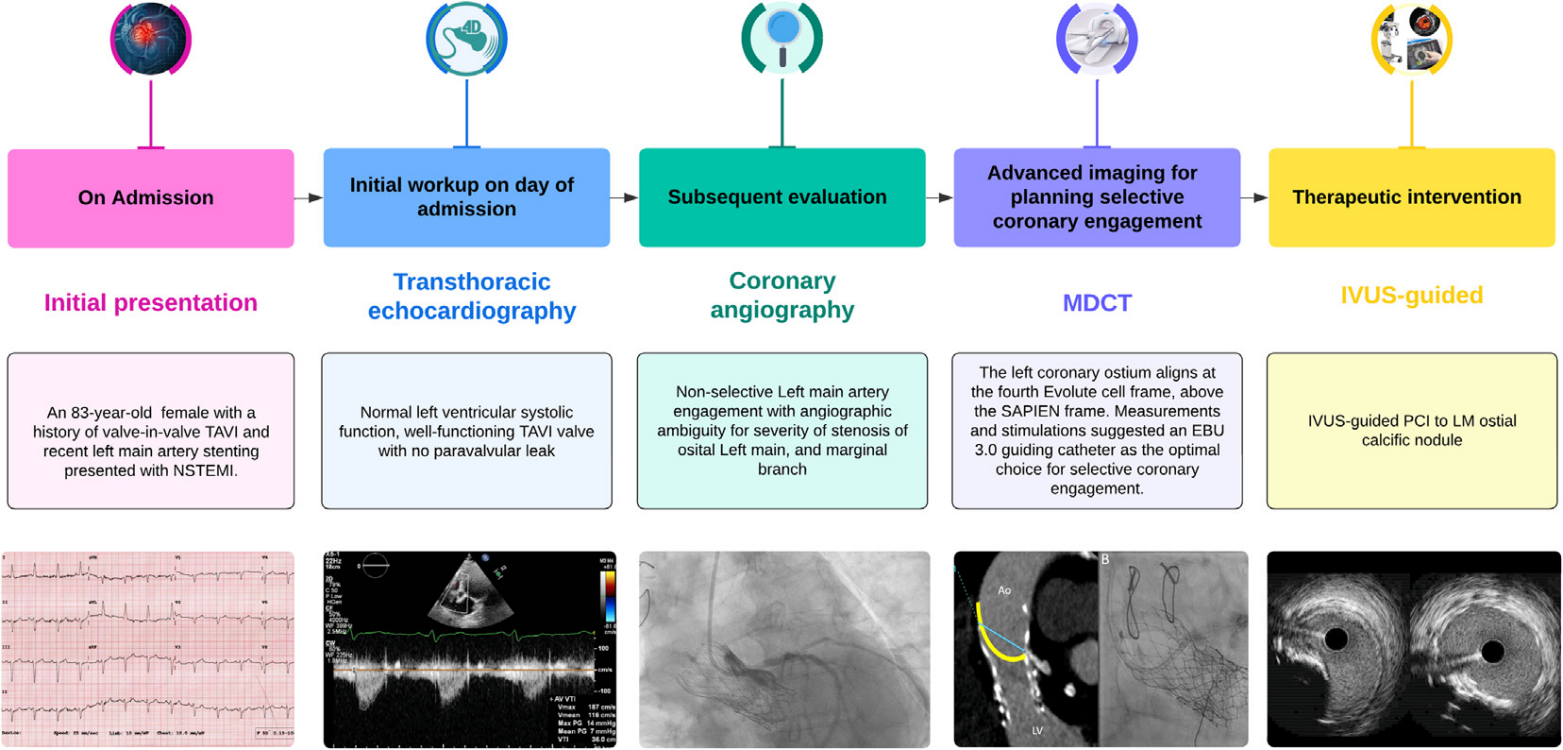
Sequential Multimodality Imaging in the Management of Complex Lesion Leading to Successful Revascularization AO = aorta; EBU = extra backup; IVUS = intravascular ultrasound; LM = left main; LV = left ventricle; MDCT = multidetector computed tomography; NSTEMI = non–ST-segment elevation myocardial infarction; PCI = percutaneous coronary intervention; TAVR = transcatheter aortic valve replacement.

Severe focal calcified stenosis was identified in the proximal segment of the intermediate branch. Pre-dilatation was conducted using a 2.5-mm balloon. Guideliner Teleflex–assisted PCI and stenting to the obtuse marginal artery were performed using an Onyx Frontier (Medtronic) 2.4 mm × 12 mm DES, resulting in a good final angiographic outcome (Figure 6B, Video 7). A guide extension was not required during the initial PCI procedure because the catheter successfully traversed the Evolut frame. However, the angle and calcification of the second lesion posed technical challenges that hindered stent crossing. In accordance with standard practice, a guide extension was used to provide additional support, thereby enabling successful navigation through the complex anatomy, irrespective of the THV.

Equipment used in this case is listed in Table 1.

**Table 1:** Equipment List for Multimodality Imaging for Percutaneous Coronary Intervention Post Valve-in-Valve Transcatheter Aortic Valve Replacement

Imaging • Transthoracic echocardiography (Philips Healthcare EPIQ CVx) ○ X5-1 probe • Multidetector computed tomography (Toshiba, 640-slice)
Percutaneous coronary intervention • 6-F sheath GuideLiner Teleflex • Guiding catheters: ○ EBU (extra backup) 3.0 guiding catheter (Medtronic) • Guidewire: Runthrough 0.014-inch × 180 cm (Terumo)
Intravascular ultrasound: 3.0-F (1.00 mm) × 135 cm OptiCross (Boston Scientific). Drug-eluting stents • Xience Skypoint 4.0 mm ×x 12 mm (successful) (Abbott) • Onyx Frontier 2.5 mm × 12 mm (successful) (Medtronic)
Balloons • Postdilatation balloons: ○ 4.5 mm × 8 mm noncompliant (NC) Quantum Apex (Boston Scientific) ○ 5.0 mm × 12 mm NC Quantum Apex (Boston Scientific) • Predilatation balloons: ○ 2.5 mm × 15 mm Emerge (Boston Scientific) ○ 2.5 mm × 12 mm NC Quantum Apex (Boston Scientific) ○ 2.5 mm × 20 mm Emerge (Boston Scientific)

## Outcome and Follow-Up

The patient was successfully discharged in stable condition 4 days after PCI with aspirin, 100 mg daily for life, and dual antiplatelet therapy for at least 1 year. The patient remained asymptomatic during a 3-month follow-up period. Written informed consent was obtained from the patient for the publication of this case report and associated images.

## Discussion

To the best of our knowledge, this is the first case reporting PCI of a calcified coronary nodule post redo TAVR that was guided by MDCT and IVUS. During the initial CAG attempt, femoral access was selected because of the inability to have radial access. However, selective LM artery engagement was unsuccessful. The ability to navigate a THV to reach the coronary vessels relies not only on anatomical factors such as coronary height and STJ dimensions but also on the design and position of the THV.^1^^,^^2^ Self-expanding valves can pose technical difficulties for coronary access because of their supra-annular placement above the coronary ostia, their closed-cell frame design, and the asymmetrical skirt.^1^ Coronary access restriction is exacerbated in cases of redo TAVR because the leaflets of the first THV may be pushed upward by the newly implanted device, in addition to the constrained catheter maneuverability by the overlapping stent frames, and the higher possibility of neocommissural malalignment.^3^^,^^4^

In the present case, MDCT was performed for the detection of anatomical features that would possibly impair coronary engagement. Unlike pre-TAVR cardiac CT, there is no standardized protocol established for post-TAVR cardiac CT acquisition. It is crucial to recognize that each imaging scan must be tailored to the specific needs of the patient.^5^ A supra-annular design, a narrow STJ (<28 mm), and a small VTA (<2 mm) were identified by Buzzatti et al^6^ as potential risk factors for coronary access restriction. Additionally, a small VTC (<4 mm) has been associated with a high risk of coronary inaccessibility, according to current established criteria.^1^^,^^7^^,^^8^ In this case, MDCT imaging revealed several favorable parameters for coronary access; the high LCA take-off above the NSP of the SAPIEN valve, the wide Evolut VTA (2.5 mm), and the free node space at the LM origin. However, unfavorable parameters were also observed, such as a small VTC (2.8 mm), a narrow STJ (24.4 mm), and a calcified nodule at the LCA ostium.

The selection of the access node was based on the MDCT imaging that provided precise localization of the LCA origin in relation to the THVs, thus identifying its position at the midlevel of the fourth Evolut node—1 node above the upper border of the SAPIEN metallic frame (Figures 3A and 5B). This detailed anatomical insight further optimized coronary cannulation.

The ideal C-arm projection for LM artery cannulation was determined on the basis of post-CT analysis, which identified the LAO 40° cranial 15° projection as the most favorable. This projection enabled precise targeting of the fourth Evolut node, thereby ensuring successful cannulation. Although the en face steep right anterior oblique (RAO) cranial projection is often advantageous for catheter rotation, it was not required in this case. The LAO 40° cranial 15° view provided an optimal trajectory for direct cannulation that eliminated the need for additional angulations.

MDCT proved valuable in guiding catheter selection by enabling simulation of the anticipated path of guiding catheters in relation to the patient’s specific anatomical configuration, thus ultimately enhancing procedural planning and outcomes. Although MDCT facilitates coronary engagement after TAVR, several strategies can optimize first-attempt success. Performing an aortogram in the LAO 40° cranial 15° projection provides precise visualization of the coronary take-off and guides optimal catheter selection. Additional techniques, including catheter downsizing, coronary wire-assisted engagement, and controlled rotational adjustments in the RAO cranial projection, further enhance procedural success.

The angiographic ambiguity regarding the severity of ostial LM artery stenosis was alleviated by IVUS. This imaging modality allowed precise assessment of the previously implanted stent and enabled accurate identification of a calcified nodule encroaching on the LM lumen that was responsible for inducing severe stenosis. The value of IVUS imaging in guiding PCI of LM and calcified lesions is well established. It offers critical insights into lesion characteristics, including the extent and distribution of calcification, thereby enabling tailored lesion preparation and optimal stent sizing. Furthermore, it plays a pivotal role in assessing post-PCI outcomes, such as stent expansion and apposition. By providing detailed and accurate anatomical information, IVUS ensures comprehensive procedural planning and optimization that contribute to improved clinical outcomes.^9^^,^^10^

## Conclusions

This case emphasizes the critical role of multimodality imaging, particularly MDCT and IVUS, in managing complex coronary lesions after redo TAVR procedures. These advanced imaging techniques enabled precise preprocedural planning, ensuring successful PCI despite the patient’s challenging anatomy. The case underscores the importance of individualized imaging protocols and the value of MDCT in planning coronary interventions, especially in patients with previous TAVRs.

## Funding Support and Author Disclosures

The authors have reported that they have no relationships relevant to the contents of this paper to disclose.

## Appendix

Video 1 Color-Doppler Transthoracic Echocardiography Color Doppler transthoracic echocardiography demonstrates a well-functioning aortic transcatheter heart valve with no evidence of either paravalvular leak or significant regurgitation.

Video 2 2-Dimensional Transthoracic Echocardiography A 2-dimensional transthoracic echocardiography 4-chamber view showing normal left ventricular systolic function, no regional wall motion abnormalities, and an intact transcatheter heart valve, confirming proper valve function without structural abnormalities.

Video 3 Nonselective Left Coronary Angiography Nonselective left coronary angiography demonstrates angiographic ambiguity in assessing the severity of ostial left main stenosis and the extent of calcific stenosis in the marginal branch.

Video 4 Selective Left Coronary Cannulation Selective left coronary cannulation targeting an Evolut (Medtronic) node above the upper border of the SAPIEN (Edwards Lifesciences) frame, with successful crossing of the left main artery lesion by using a Runthrough guidewire (Terumo).

Video 5 IVUS of Left Circumflex Artery, Until Ostium of Left Main Artery Intravascular ultrasound (IVUS) of the left circumflex artery extending to the ostium of the left main artery, revealing a well-patent stent in the left main artery and proximal left circumflex artery. The ostium of the left main artery, however, is uncovered and shows 90% calcified nodular stenosis.

Video 6 Final IVUS Post-Stenting of Ostial Left Main Artery Final intravascular ultrasound (IVUS) imaging post-stenting of the ostial left main artery showing proper stent expansion and absence of strut malapposition.

Video 7 Final Left Coronary Angiography Post-Revascularization Final left coronary angiography showing good angiographic outcome in both ostial left main artery and proximal segment of the intermediate branch.
